# Optical computing for optical coherence tomography

**DOI:** 10.1038/srep37286

**Published:** 2016-11-21

**Authors:** Xiao Zhang, Tiancheng Huo, Chengming Wang, Wenchao Liao, Tianyuan Chen, Shengnan Ai, Wenxin Zhang, Jui-Cheng Hsieh, Ping Xue

**Affiliations:** 1 State Key Laboratory of Low-dimensional Quantum Physics and Center for Atomic and Molecular Nanoscience, Department of Physics, Tsinghua University and Collaborative Innovation Center of Quantum Matter, Beijing 100084, China

## Abstract

We propose an all-optical Fourier transformation system for real-time massive data processing in high speed optical coherence tomography (OCT). In the so-called optical computing OCT, fast Fourier transformation (FFT) of A-scan signal is optically processed in real time before being detected by photoelectric detector. Therefore, the processing time for interpolation and FFT in traditional Fourier domain OCT can be dramatically eliminated. A processing rate of 10 mega-A-scans/second was experimentally achieved, which is, to our knowledge, the highest speed for OCT imaging. Due to its fiber based all-optical configuration, this optical computing OCT system is ideal for ultrahigh speed volumetric OCT imaging in clinical application.

OCT is a depth-resolved biomedical *in vivo* imaging technique providing cross-sectional and three-dimensional images of tissue microstructure with resolution up to a few micrometers^1^,^2^,^3^. In the past few decades, OCT has been applied in a wide range of applications including clinical and material research areas. For some biomedical applications such as surgical guidance, real-time volumetric OCT (4D-OCT) imaging is in high demand. At present, the main bottlenecks for 4D-OCT are real-time data acquisition and data processing, e.g. interpolation, FFT. For example, ultrafast frequency domain optical coherence tomography (FD-OCT) using a discrete spectrometer may be developed with A-scan rates of 2.5~10 MHz^4^. However, the data processing speed is only 1.02 mega-A-scans/second even if using field programmable gate arrays (FPGAs)^4^, which is far below the requirement of 4D-OCT. Thanks to the graphics processing unit (GPU) technology, the processing speeds of several mega-A-scans/second may be achieved under different algorithms including linear/cubic spline interpolation with the FFT, Non-uniform FFT, linear/cubic spline interpolation with the FFT and numerical dispersion compensation^5^. With a combination of a fast swept laser source, dual high-end digitizer cards and a state of the art GPU card with dedicated processing software, 3.2 MHz A-scan rate/processing speed was demonstrated for high definition 4D-OCT *in vivo*^6^. However, for high definition volumetric imaging in real time, i.e., 1000 pixels along 3 spatial dimensions and 30 volumetric imaging per second, the data flow will be 1000 × 1000 × 1000 × 30 = 30 GVoxel/s. To process these massive data is a big challenge to the performance of current GPUs and CPUs.

Optical computing^7^,^8^,^9^,^10^,^11^ (OC) uses photons for computation and enables ultrafast information processing^12^,^13^,^14^. It may play an important role in solving the big challenge of massive data processing applications in optical imaging, because photons in bio-imaging may be possibly processed directly with all-optical processing system. In 4D-OCT, the massive image data come from the interpolation + FFT in spectral-domain other than in time-domain. Therefore, if one can find a way to implement the optical processing of optical spectrum, the big challenge of massive data processing by GPUs and CPUs may be solved optically. In this paper we propose a novel optical computing technique to process the signal in spectral-domain with fiber-optics system other than compute interpolation + FFT with electronic computer, resulting in great enhancement of processing time and hence imaging speed as well.

## Theory

The optical computing technique is based on an all-optical real-time Fourier transformation system. As we know, the *α*-angle fractional Fourier transformation of a function *f (t*) is defined by the integral^15^


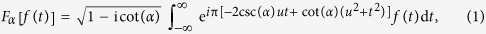


where *α* is real and *u* is the variable in the fractional domain. For *α* = π/2, the fractional Fourier transformation of *f (t*) is equal to the ordinary Fourier transformation of *f (t*):


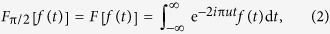


where *F* [ ] indicates the ordinary Fourier transformation and *u* becomes the variable in the frequency domain.

Equation (1) can be re-written as


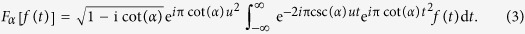


Here, we define a scaled Fourier transformation *F*′ [ ]:


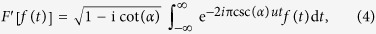


where the scale factors in frequency domain and intensity are determined by *α*.

According to equations (3) and (4), the following relationship between the fractional Fourier transformation and the scaled Fourier transformation is obtained:


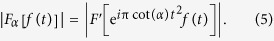


Let the function *f (t*) have a width of Δ*t*. When |*t*| > Δ*t*/2, *f (t*) = 0. When |*t*| ≤ Δ*t*/2, the maximum of *t*^2^ is Δ*t*^2^/4. If *α* is close to π/2 enough to satisfy the approximate condition of πcot(*α*)Δ*t*^2^/4 ≪ 2π, the term exp[iπcot(*α*)*t*^2^] ≈ 1 in equation (5). Therefore the intensity of fractional Fourier transformation is equal to the intensity of scaled Fourier transformation


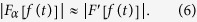


**Figure 1:**
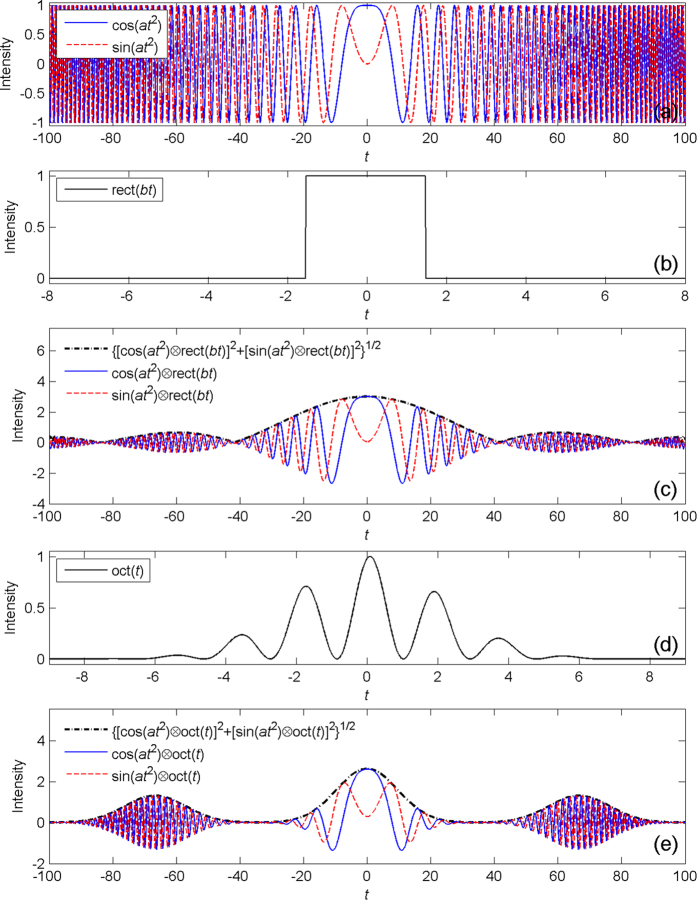
Calculation of the approximate Fourier transformation by equation (9). (**a**) The waveforms of cos(*at*^2^) and sin(*at*^2^). (**b**) The waveform of rect(*bt*). (**c**) The convolution of cos(*at*^2^) and rect(*bt*), the convolution of sin(*at*^2^) and rect(*bt*), the square root of sum of the squares of them. (**d**) The waveform of oct(*t*). (**e**) The convolution of cos(*at*^2^) and oct(*t*), the convolution of sin(*at*^2^) and oct(*t*), the square root of sum of the squares of them.

By re-writing equation (1), the fractional Fourier transformation of *f (t*):


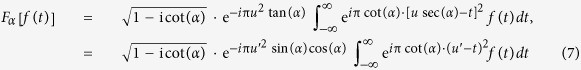


where *u*′ = *u*·sec(*α*). Notice that the integral in equation (7) is in the form of convolution. Thus, the intensity of fractional Fourier transformation of *f (t*)^16^:


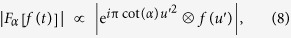


where ⊗ denotes a convolution and ∝ proportional to. According to equation (8), if only taking into account the relative intensity of fractional Fourier transformation of *f (t*), the convolution can be used instead of fractional Fourier transformation. Let *a *= πcot(*α*), a time-domain intensity of fractional Fourier transformation of *f (t*) can be written as


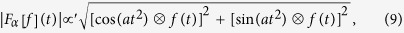


where ∝′ denotes the similarity of two functions, indicating that one can be obtained from the other by scaling in amplitude and/or variable. When using the equation above, it is clear that we have to compute convolution twice. Let us take *f (t*) = rect(*bt*) as an example to demonstrate how to get the approximate Fourier transformation of *f (t*) by equation (9), where rect() is the rectangle function. Here let *a* = 0.025, *b* = 1/3. The waveforms of cos(*at*^2^), sin(*at*^2^) and rect(*bt*) are shown in Fig. 1(a,b). For *a·*Δ*t*^2^/4 ≈ 0.056 ≪ 2π, the approximate condition is satisfied. It is interesting to see that the waveform of {[cos(*at*^2^) ⊗ rect(*bt*)]^2^ + [sin(*at*^2^) ⊗ rect(*bt*)]^2^}^1/2^ in Fig. 1(c) is very close to the sinc function, which is known as the Fourier transformation of rectangle function.

Due to cos^2^ + sin^2^ = 1, the square root of sum of the squares of cos(*at*^2^) ⊗ rect(*bt*) and sin(*at*^2^) ⊗ rect(*bt*) is a slowly varying function, though both of them are fast varying functions. In addition, Fig. 1(c) shows clearly that the waveform of {[cos(*at*^2^) ⊗ rect(*bt*)]^2^ + [sin(*at*^2^) ⊗ rect(*bt*)]^2^}^1/2^ is the slowly varying envelope of cos(*at*^2^) ⊗ rect(*bt*) or sin(*at*^2^) ⊗ rect(*bt*). For this reason, it is not necessary to compute convolution twice to get the relative intensity of fractional Fourier transformation of *f (t*) and equation (9) can be improved further:


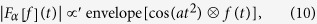


where the envelope function is used to get the slowly varying envelope of a fast varying function. To test equation (10) once more, we let *f (t*) = oct(*t*), where oct(*t*) is the basic signal to be Fourier transformed in OCT and mathematically expressed as oct(*t*) = [cos(*ct*) + 1]/2·exp(−*t*^2^/*d*^2^), as shown in Fig. 1(d). Taking *a* = 0.025, *c* = 3.3 and *d* = 3, we get *a·*Δ*t*^2^/4 ≈ 0.156 ≪ 2π so that the approximate condition is satisfied. Again, it is clear that the waveform of {[cos(*at*^2^) ⊗ oct(*t*)]^2^ + [sin(*at*^2^) ⊗ oct(*t*)]^2^}^1/2^ is the slowly varying envelope of cos(*at*^2^) ⊗ oct(*t*) or sin(*at*^2^) ⊗ oct(*t*), as shown in Fig. 1(e). Likewise, the waveform of {[cos(*at*^2^) ⊗ oct(*t*)]^2^ + [sin(*at*^2^) ⊗ oct(*t*)]^2^}^1/2^is very similar to the Fourier transformation of oct(*t*), which is in form of gauss(*u − c*/2π) + gauss(*u* + *c*/2π) + 2·gauss(*u*), where gauss(*u*) is the Gaussian function. In practice, all OCT signals to be Fourier transformed can be regarded as the linear combinations of oct(*t*) with different parameters, so the equation (10) is definitely suitable for generalized OCT signals.

Under the approximate condition of πcot(*α*)Δ*t*^2^/4 ≪ 2π, according to equations (6) and (10), we find


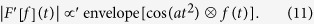


It means that the slowly varying envelope of convolution of *f (t*) and cos(*at*^2^) is similar to the intensity of Fourier transformation of *f (t*). Equation (11) is therefore the fundamental principle of all-optical real-time Fourier transformation system. Interestingly, this principle can be used in processing OCT data since only the relative intensity of Fourier transformation of spectral energy density of light signal is necessary for OCT imaging.

In optical computing OCT (OC-OCT or OC^2^T) system, we utilized time-spectrum convolution (TSC) for real-time computing of convolution. The TSC process means that the intensity waveform of light output by a first-order dispersive medium with a spectral filtering is determined by the convolution of the input light temporal profile and the light energy spectrum scaled along the time-domain according to the group-delay curve of the dispersive medium^17^,^18^. Mathematically,


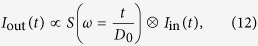


where *t* is the time variable, *I*_out_(*t*) the intensity waveform of light output by a first-order dispersive medium with a spectral filtering, *I*_in_(*t*) the input light temporal profile, *ω* the base-band radial frequency variable referred to the central frequency of the light source, *S(ω*) the spectral energy density of the incoherent broadband light after spectral filtering, *D*_0_ the group velocity dispersion of first-order dispersive medium, defined as the slope of the group delay, and *S(ω* = *t/D*_0_) the time-domain scaled version of spectral energy density using a frequency-to-time mapping law determined by the dispersive medium *ω* = *t/D*_0_.

**Figure 2:**
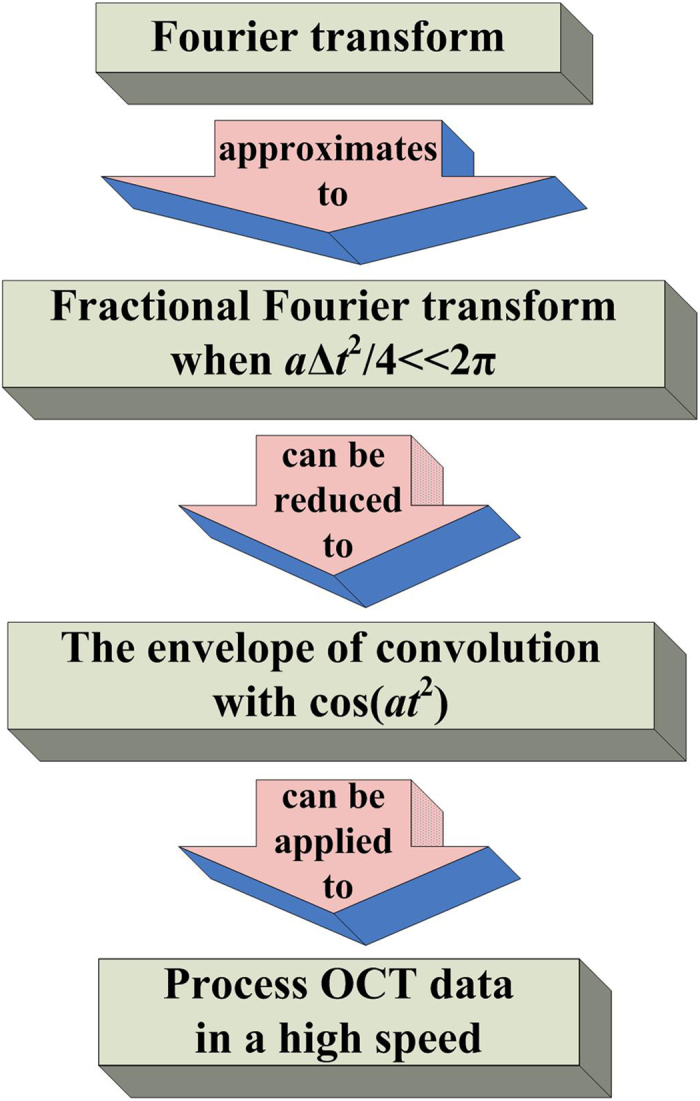
The fundamental principle of OC^2^T.

The fundamental principle of OC^2^T in this paper is shown in Fig. 2. Mathematically, under the approximate condition, the intensity of Fourier transformation equals approximately the intensity of fractional Fourier transformation. If only interested in relative intensity of fractional Fourier transform of a certain function *f (t*), the intensity of fractional Fourier transformation of *f (t*) can be re-written in the form of envelope of convolution of *f (t*) and cos(*at*^2^), where *a* is a constant. In practice, we utilized TSC concept for real-time computing of convolution and it is used to process the massive 3D OCT image data in real time as a unique technology in OC^2^T.

## Experimental results

**Figure 3:**
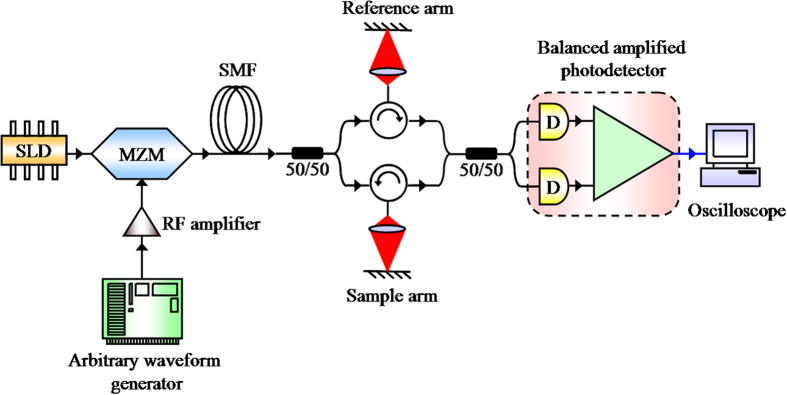
Schematic of the experimental setup of OC^2^T. SLD: superluminescent diode, MZM: Mach–Zehnder modulator, SMF: single mode fiber, RF: radio frequency.

The experimental setup of OC^2^T is shown in Fig. 3 (see also Methods). With TSC parameters of *D*_0_ = 130 ps^2^ (~102 ps/nm), *a* = 0.15 ns^−2^ and Δ*λ* = 40 nm, the approximate condition *a*Δ*t*^2^/4 ≪ 2π is satisfied, where Δ*t* = ~4 ns. As described before, this means that the experimental setup in Fig. 3 acts as a real-time Fourier transformation system for the spectrum of OCT interferometer. Moreover, the spectrum to be Fourier transformed is actually a function of *ω* in frequency domain. As a result, the interpolation in the wavenumber domain and FFT, which are essential in traditional FD-OCT, are no longer necessary in OC^2^T, since these two steps are optically finished in real time before the light signal is converted to electrical signal by balanced photodetector. It is worth to note that the *ω* ~ *t* relation here is exactly the same as *k* ~ *z* relation in FD-OCT.

**Figure 4:**
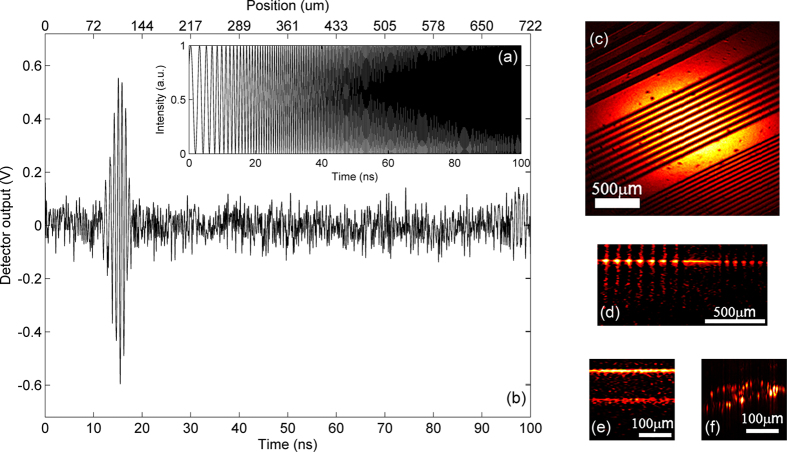
Experimental results. (**a**) A single period of the cos(*at*^2^) waveform data used in AWG. (**b**) A single period of the waveform measured by AC-coupled balanced amplified photodetector. (**c**) The *en-face* OC^2^T image of a variable-frequency resolution target. (**d**) The image of a variable-frequency resolution target. (**e**) The image of a cover glass. (**f**) The image of onion layer.

In experiment, the scalable function of a single period (100 ns) of the cos(*at*^2^) waveform was generated by arbitrary waveform generator (AWG) with its highest frequency of ~5 GHz, as shown in Fig. 4(a). Here, the A-scan rate is 1/*T*_0_ = 1/(100 ns) = 10 MHz, where *T*_0_ is the period of the cos(*at*^2^) waveform. So the A-scan rate can be tuned by changing the value of *T*_0_ with AWG. All the radio frequency (RF) cables employed in the system can support up to 18 GHz RF signal with low loss. Only taking into account the positive frequency part of the Fourier transformation of the spectrum for OCT imaging, we neglected the negative frequencies and zero frequency, as shown in Fig. 1(a), to halve the A-scan time and thus double the A-scan rate/data process speed.

We used a flat mirror as a sample in the experiment to demonstrate the feasibility of real-time Fast Fourier transformation (FFT) of A-scan signal with optical computing. As we know, the positive frequency part of Fourier transformation of the spectrum output by OCT interferometer is theoretically in the form of *Γ(z*) ⊗ *δ(z* − Δ*l*), where *z* is the variable in the space domain and Δ*l* the optical path difference between reference arm and sample arm. *Γ(z*) is Gaussian because of the Gaussian spectrum of superluminescent diode (SLD). As discussed before, because real-time FFT of A-scan signal was already implemented via the optical system, the temporal envelope of the signal measured by photodetector represented the position of the mirror, which was dramatically observed in experiment, as shown in Fig. 4(b). The oscillating signal with Gaussian envelope was actually the resultant signal measured by AC-coupled balanced amplified photodetector. By adjusting the position of the mirror, the center of the Gaussian envelope of the signal changed accordingly. (Please see Supplementary Video 1). The video clearly shows the real-time FFT processing to achieve the sample’s structural signal with no time delay. Obviously, this proved the first demonstration of real-time FFT of A-scan signal with optical computing for OCT. In Fig. 4(b), maximum imaging range and axial resolution of OC^2^T are ~722 μm and ~38 μm. Just like the traditional FD-OCT, the signal frequency of optical computing OCT system get higher as imaging depth increases, while its highest frequency is limited by the arbitrary waveform generator. So the maximum image range is determined by the bandwidth of arbitrary waveform generator. By changing the maximum frequency of waveforms with arbitrary waveform generator, the image range could be tuned.

Then we installed a raster scanning system with an 8.1 kHz resonant scanner for fast scanning axis and a 13.5 Hz galvanometer scanner for slow scanning axis in the sample arm of OCT system. Thus, this OC^2^T system for real-time imaging was set to scan at a frame rate of 16.2 kHz and a volume rate of 27 Hz. Moreover, the lateral and axial resolutions are ~33 μm and ~38 μm, respectively with ~50 dB signal-to-noise ratio and ~240 μm @-3dB roll-off. The system has an A-scan rate of 10 MHz and one frame typically contains ~200 A-scans in the experiment. The *en-face* and cross-sectioning imaging of a variable-frequency resolution target are shown in Fig. 4(c,d), respectively, with clear structures of different spatial frequencies. Cross-sectioning imaging of a cover glass and onion layer is also implemented, as shown in Fig. 4(e,f), respectively. The interfaces of the cover glass and cellular structure of onion are also clearly distinguishable. The system SNR may decrease as A-scan rate increases, because A-scan period gets shorter, leading to higher signal frequency and wider detection bandwidth. The system sensitivity is relatively low due to the inverse proportional relation of the sensitivity and bandwidth, which is as wide as ~20 GHz in our system. The single mode fiber (SMF28, Corning) used in our experiment is not an ideal linearly chirped dispersive medium required by time-spectrum convolution process and therefore may also lead to the degradation of signal and thus the sensitivity as well. As a result, the axial resolution also gets worse than expected. To improve the SNR, higher light power could be employed and a frequency mixer can be used to lower down the detection bandwidth. Utilizing an ideal linearly chirped dispersive medium such as customized linearly chirped fiber Bragg grating in time-spectrum convolution process will also enhance the system sensitivity and the axial resolution as well.

With our current optical computing system, the OCT operated at a 10 MHz A-scan rate/A-scan processing speed, which is, to the best of our knowledge, the fastest speed for OCT imaging. Because all the signal is optically processed in real time, one feature of OC^2^T is that its A-scan processing speed equals to A-scan rate determined by the waveform generated by AWG, which may be even faster although is currently limited by the electronics we have in the lab. Furthermore, due to its all-optical configuration, OC^2^T has the advantage of being much more stable, compact and convenient. Note that the fundamental principle of optical computing technique is independent on wavelength, so it can be definitely applied to any other wavelength bands such as 800 nm and 1300 nm. Compared to the expensive fast swept laser in high speed SS-OCT, optical computing OCT also need utilize an expensive 5 GHz arbitrary waveform generator. However, only the waveform of cos(*at*^2^) is needed and the arbitrary waveforms are not necessary for optical computing OCT. Therefore, a fixed waveform generator of cos(*at*^2^) may be designed to lower down the system cost.

## Conclusions

In summary, we report a novel optical computing technique for high speed real-time OCT, as called OC-OCT or OC^2^T for the first time. Different from traditional FD-OCT, the interpolation in the wavenumber domain and FFT by GPU or CPU are not needed any more in OC^2^T. Instead of software algorithm and GPUs computing, we employed an all-optical real-time Fourier transformation system to process the A-scan data before the OCT light signal is converted to electrical signal by the photodetector. Using this optical computing method, the A-scan rate and A-scan processing speed of OC^2^T have reached 10 mega-A-scans/second in our experiment. To our knowledge, this OC^2^T is the fastest real-time sampling and processing OCT system to date. Furthermore, OC^2^T based on the all-fiber configuration is much more stable and easier to be used. The OC^2^T is able to be fast enough to achieve the video rate 4D-OCT imaging at a large volume size and therefore further enable the real-time virtual reality, which is of great value in surgical guidance. We believe that this new technology will initiate a new way of ultra-fast OCT imaging.

## Methods

As shown in Fig. 3, low coherence CW light centered at 1550 nm with average power of 22 mW and bandwidth of 40 nm was generated by superluminescent diode (SLD). The CW light was modulated by a 10 GHz bandwidth intensity modulator, known as Mach–Zehnder modulator (MZM). The MZM was biased by a power supply and driven by a cos(*at*^2^) waveform signal generated by an arbitrary waveform generator (AWG). A radio frequency (RF) amplifier with adjustable gain was used between AWG and MZM as a modulator driver to reach the typical RF drive voltage of 5.5 V for MZM. As first-order dispersive medium and spectral filter in TSC, a 6.5 km long single mode fiber (SMF) and OCT interferometer were employed after MZM. The two outputs of interferometer were detected by an AC-coupled balanced amplified photodetector which was able to remove the common-mode noise and improve signal-to-noise ratio of the system. Finally, DC-free waveform was shown in an 8 GHz bandwidth oscilloscope. In fact, the SMF used as the first-order dispersive medium can be replaced by linearly chirped fiber Bragg grating (LCFBG) which is more compact and with low loss. LCFBG has an advantage that its dispersion, linearity of dispersion and other properties may be specially designed to behave better than fiber.

## Additional Information

**How to cite this article**: Zhang, X. *et al.* Optical computing for optical coherence tomography. *Sci. Rep.* **6**, 37286; doi: 10.1038/srep37286 (2016).

**Publisher’s note**: Springer Nature remains neutral with regard to jurisdictional claims in published maps and institutional affiliations.

## Supplementary Material

Supplementary Information

Supplementary Video 1

## References

[b1] Huang D. *et al.* Optical coherence tomography. Science 254, 1178–1181, 10.1126/science.1957169 (1991). 1957169 PMC4638169

[b2] Drexler W. *et al.* Ultrahigh-resolution ophthalmic optical coherence tomography. Nat. Med. 7, 502–507, 10.1038/86589 (2001). 11283681 PMC1950821

[b3] Zhang N. *et al.* Compressed sensing with linear-in-wavenumber sampling in spectral-domain optical coherence tomography. Opt. Lett. 37, 3075–3077, 10.1364/OL.37.003075 (2012). 22859090

[b4] Choi D., Hiro-Oka H., Shimizu K. & Ohbayashi K. Spectral domain optical coherence tomography of multi-MHz A-scan rates at 1310 nm range and real-time 4D-display up to 41 volumes/second. Biomed. Opt. Express 3, 3067–3086, 10.1364/BOE.3.003067 (2012). 23243560 PMC3521307

[b5] Zhang K. & Kang J. U. Graphics processing unit-based ultrahigh speed real-time Fourier domain optical coherence tomography. IEEE J. Sel. Top. Quantum Electron. 18, 1270–1279, 10.1109/JSTQE.2011.2164517 (2012).

[b6] Wieser W. *et al.* High definition live 3D-OCT *in vivo*: design and evaluation of a 4D OCT engine with 1 GVoxel/s. Biomed. Opt. Express 5, 2963–2977, 10.1364/BOE.5.002963 (2014). 25401010 PMC4230855

[b7] Miller A. Optical computing: Elements of an optical engine. Nature 323, 13–14, 10.1038/323013a0 (1986).

[b8] Woods D. & Naughton T. J. Optical computing: Photonic neural networks. Nature Phys. 8, 257–259, 10.1038/nphys2283 (2012).

[b9] Caulfield H. J. & Dolev S. Why future supercomputing requires optics. Nature Photon. 4, 261–263, 10.1038/nphoton.2010.94 (2010).

[b10] Solli D. R. & Jalali B. Analog optical computing. Nature Photon. 9, 704–706, 10.1038/nphoton.2015.208 (2015).

[b11] Fabre C. Optical computing: The optical Ising machine. Nature Photon. 8, 883–884, 10.1038/nphoton.2014.292 (2014).

[b12] Cotter D. *et al.* Nonlinear optics for high-speed digital information processing. Science 286, 1523–1528, 10.1126/science.286.5444.1523 (1999). 10567251

[b13] Marandi A., Wang Z., Takata K., Byer R. L. & Yamamoto Y. Network of time-multiplexed optical parametric oscillators as a coherent Ising machine. Nature Photon. 8, 937–942, 10.1038/nphoton.2014.249 (2014).

[b14] Ferrera M. *et al.* On-chip CMOS-compatible all-optical integrator. Nature Commun. 1, 29, 10.1038/ncomms1028 (2010). 20975692 PMC2982162

[b15] Ozaktas H. M., Zalevsky Z. & Kutay M. A. The Fractional Fourier Transform with Applications in Optics and Signal Processing 118–122 (Wiley, 2001).

[b16] Cuadrado-Laborde C., Carrascosa A., Díez A., Cruz J. L. & Andres M. V. Photonic fractional Fourier transformer with a single dispersive device. Opt. Express 21, 8558–8563, 10.1364/OE.21.008558 (2013). 23571945

[b17] Park Y. & Azaña J. Ultrahigh dispersion of broadband microwave signals by incoherent photonic processing. Opt. Express 18, 14752–14761, 10.1364/OE.18.014752 (2010). 20639961

[b18] Park Y. & Azaña J. Optical signal processors based on a time-spectrum convolution. Opt. Lett. 35, 796–798, 10.1364/OL.35.000796 (2010). 20237602

