# Supplementary material for: Optical computing for optical coherence tomography
Source: Sci Rep. 2016 Nov 21;6:37286. doi: 10.1038/srep37286 (PMC5116674; doi:10.1038/srep37286)
Supplement: Supplementary Information [file srep37286-s1.doc]

**Optical computing for optical coherence tomography: supplementary material**

**Xiao Zhang, Tiancheng Huo, Chengming Wang, Wenchao Liao, Tianyuan Chen, Shengnan Ai, Wenxin Zhang, Jui-Cheng Hsieh & Ping Xue^*^**

State Key Laboratory of Low-dimensional Quantum Physics and Center for Atomic and Molecular Nanoscience, Department of Physics, Tsinghua University and Collaborative Innovation Center of Quantum Matter, Beijing 100084, China.

*xuep@tsinghua.edu.cn

**Supplementary video 1**

The video shows the screen record of oscilloscope and the tuning of optical path difference of interferometer (inset) at the same time. The time interval between two markers is 100ns. By adjusting the optical path difference of interferometer, the center of the Gaussian envelope of the signal changed accordingly. This video clearly shows the real-time FFT processing of optical spectrum to achieve the sample’s structural signal. Obviously, it proved the first demonstration of real-time FFT of A-scan signal with optical computing for OCT.
